# Responses of Soil Microbial Communities Associated with Phosphorus Transformation to Land-Use Alternations in a Meadow Grassland, Northeast China

**DOI:** 10.3390/microorganisms13030624

**Published:** 2025-03-08

**Authors:** Li Yu, Ying Zhang, Zhenbo Cui, Chengyou Cao

**Affiliations:** 1 College of Life and Health Sciences, Northeastern University, Shenyang 110169, China; yuli@mail.neu.edu.cn (L.Y.); zhangying@mail.neu.edu.cn (Y.Z.); cuizhenbo@mail.neu.edu.cn (Z.C.); 2 Liaoning Province Key Laboratory of Bioresource Research and Development, Northeastern University, Shenyang 110169, China

**Keywords:** *gcd*, inorganic phosphorus solubilization, land-use type, organic phosphate mineralization, *phoD*

## Abstract

Land-use changes in meadow grasslands in semi-arid areas usually significantly affect soil environment and microbiota. However, studies on the response of soil P-cycle-related microbial communities to land-use conversions are still limited. In this study, a series of land-use types including upland field, paddy field, poplar plantation, and their adjacent natural meadow grassland in the Horqin Sandy Land of Northeast China were selected, and the diversities and structures of soil microbial communities involved in organic P mineralization (*phoD*-harboring community) and inorganic phosphate solubilization (*gcd*-harboring community) were investigated by the high-throughput sequencing technique. Land-use type had significant influences on soil physicochemical properties, enzymatic activities, and P conversion rates, thereby altering the structures of soil *gcd* and *phoD* communities. Soil *phoD* microbes are more abundant and have more contributions to available P than *gcd* microbes. The responses of *gcd* or *phoD* communities to land-use type were characterized as the quantitative shift in the relative abundance of dominant taxa; however, the basic compositions of the two communities were slightly affected. Soil pH, EC, and nutrient contents (including organic matter and total and available N, P, and K) all significantly affected soil *gcd* and *phoD* microbial communities. The abundance of *phoD* and *gcd* genes varied with land-use type and could be used as indicators for estimating the bioavailability of soil P.

## 1. Introduction

The increase in the global population and rapid economic development have led to significant alternations in land cover and land-use types, which have become a prominent issue of global environmental change. These changes directly or indirectly impact the structure and function of ecosystems, thereby triggering a cascade of environmental responses [1,2]. Intensive soil perturbations can alter intrinsic soil properties and the structures of dwelling microbial communities in local ecosystems. Therefore, land-use conversion is an important factor affecting soil quality. Thus, ecological processes in the conversions should be elucidated to predict the development and sustainability of ecosystem services. The Horqin Sandy Land was formerly a sparse forest grassland landscape in Northeast China. However, in recent decades, a large area of the natural meadow grasslands has been reclaimed as farmlands due to the increase in the population of local residents. This phenomenon can significantly affect the landscape pattern and functions of grassland ecosystems. Land-use conversion can alter the input of organic matter, including the litter of aboveground vegetation and underground roots, thus affecting soil carbon (C) and nitrogen (N) storage potentials [3]. Soil microorganisms can sensitively respond to environmental variations, and many studies have examined the changes in the microbial communities under different land uses [4,5]. Wang et al. [6] reported that soil microbial communities played more important roles in driving the changes in C and N cycles induced by land-use conversion. However, few studies have focused on the effect of land-use conversion on soil phosphorus (P) transformation, and the variations in the diversity and structure of soil P-cycle-related microbial community during land-use conversion are still unknown. Considering the increasing reclamation activities in native meadow grasslands in Northeast China, studies on the responses of the diversities and structures of soil microbial communities involved in P transformation to land-use conversion should be performed to gain a further understanding of the microbiological mechanism of the soil P-cycling process.

As an essential nutrient element for all living cells, P is one of the major limiting nutrients for plant growth in ecosystems [7]. P exists in soil in organic and inorganic forms. Inorganic P includes residual primary minerals, secondary inorganic phosphates, etc., while organic P mainly comes from soil organic matter, plants, microbial residues, and organic fertilizers [8]. Only inorganic orthophosphate ions in soil solution can be absorbed and utilized by plants [8]; therefore, it is a necessary process converting organophosphate into its inorganic form for P assimilation. This process is mainly catalyzed by soil phosphatases, which are secreted by microbes or plant roots. Meanwhile, phosphate-solubilizing bacteria can secrete various organic acids and facilitate the conversion of P from an unavailable form to soluble forms [9,10]. Phosphatase is the main enzyme in the soil organophosphorus cycle, consisting of alkaline phosphatase (ALP) and acid phosphatase (ACP). In many soil types, ALP and ACP activities have been extensively studied to assess organic phosphate mineralization under different agricultural management regimes [11]. Studies have shown that ALP entirely comes from soil microorganisms and is an essential enzyme for organic phosphate mineralization [12]. It has been confirmed that alkaline phosphatase-encoding genes are divided into three different families based on their sequence similarity and substrate specificity, namely, *phoA*, *phoD*, and *phoX* [13]. *phoA* is one of the earliest studied phosphomonopolylipases, which uses zinc and magnesium as cofactors. Both *phoX* and *phoD* use calcium as a cofactor and hydrolyze mono- and diesters of phosphate [14,15]. Analysis of the prevalence of three different ALP families (*phoA*, *phoD*, and *phoX*) in metagenomic datasets showed that *phoD* was the most common ALP in soil samples and was usually selected as the most representative ALP in soil bacterial communities [16].

In inorganic phosphorolytic metabolism, the Quinoprotein glucose dehydrogenase (GDH) is the key enzyme to catalyze glucose to gluconic acid with pyrroloquinoline quinone (PQQ) as a coenzyme. GDH is encoded by the *gcd* gene. The expression of the *gcd* gene is gradually inhibited as the concentration of soluble phosphate increases; therefore, it is inferred that the regulation of *gcd* gene transcription by soluble phosphate may be the basis for the regulation of gluconic acid secretion, thus regulating the solubilization activity of phosphate [17]. The diversity and abundance of *gcd* genes are significantly correlated with environmental factors such as dissolved oxygen, P hydrochloride, and dissolved total P; the composition of *gcd*-harboring microbial communities and their distributions are significantly correlated with dissolved oxygen, total organic C, and dissolved total P [18]. Up to the present, studies focusing on *gcd*-specific bacteria have been limited.

In this study, we investigated and compared the diversities and the structures of soil *gcd*- or *phoD*-harboring microbial communities in an adjacent poplar plantation, dry land, and paddy field, which were all transformed from a meadow grassland 20 years ago in the Horqin Sandy Land, by using the high-throughput sequencing technique. The purposes of this study were to (1) obtain the diversities of *phoD*- and *gcd*-carrying microbes in this meadow soil, (2) compare the differences in the response of *phoD* or *gcd* microbial communities to land-use type; and (3) discuss the relationships among soil properties, P transformation function, and the structures of the two microbial communities. We hypothesized that land-use conversion significantly alters the fraction composition and concentration of soil P, and the effects are significantly correlated with the responses of *phoD*- and *gcd*-carrying microbial communities. The results are expected to enrich the microbial diversities involved in P transformation function in meadow soil and offer some novel perspectives on the sustainability evaluation of land-use systems in semi-arid areas.

## 2. Materials and Methods

### 2.1. Study Location and Site Description

**Figure 1:**
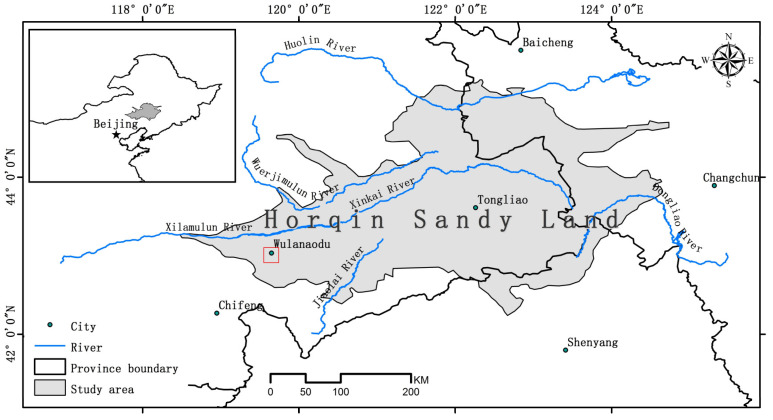
Study location (geographical location map was provided by Dr. Renhui Miao).

This study was conducted in the desertification control experimental demonstration areas of the Wulanaodu Desertification Combating Research Station (43°02′ N, 119°39′ E) under the Institute of Applied Ecology, Chinese Academy of Sciences, the west of Horqin Sandy Land, Northeast China (Figure 1). The Wulanaodu region has a typical semi-arid continental monsoon climate. The annual average wind speed, temperature, and precipitation were 4.5 m s^−1^, 6.3 °C, and 340.5 mm, respectively. The original vegetation was elm steppe–woodland [19], which belongs to the Mongolian flora. The representative species include *Aneurolepidium chinense*, *Cleistogenes chinensis*, *Arundinella hirta*, *Adenophora tetraphylla*, *Calamagrostis epigeios*, etc. Large areas of natural meadow grasslands are distributed in the Wulanaodu region, which support the development of local animal husbandry. However, the meadow grasslands have been extensively cultivated in recent decades to meet more income needs. At present, a mosaic of meadow grassland, upland fields, paddy fields, and forest plantation has been gradually formed in this region, which has provided better experiment sites to study the response of soil microbial communities to land-use type.

### 2.2. Experimental Design and Soil Sampling

Soils were sampled in October 2021. A series of land-use types including an upland field, a paddy field, a poplar plantation, and their adjacent natural meadow grassland (designated as UF, PF, PP, and NMG, respectively) were selected as experimental sites. UF, PF, and PP all originated from NMG, and the soil types are the same and classified as alkali meadow soil. The utilization times or plantation ages were all 20 years. UF and PF usually plant *Zea mays* L. and *Oryza sativa* L., respectively. PF and UF were annually fertilized using urea, (NH_4_)_2_HPO_4_, and KCl during maize seeding, rice transplanting, and crop growth. The averages of height and diameter at breast of the poplars at the PP site were 14.5 m and 16.4 cm, respectively. Three fields of UF, PF, PP, and NMG were selected as experimental sites, respectively, and each site was 300 cm away from the other one. In each site, one plot (30 m × 30 m) was set up for sampling. In each plot, 20 subsamples (0–10 cm) were randomly collected and pooled as one sample. All the samples were sieved in the field using a 2 mm screen. Half of each sample was stored in a −80 °C refrigerator after vacuum freeze-drying for DNA extraction, and the other half was air-dried for soil property determinations.

### 2.3. Soil Property Determinations

Soil moisture (SM) was determined by the drying method. Soil pH and electrical conductivity (EC) were determined in 1:2.5 and 1:5 soil–water suspensions, respectively. Soil available P (AP) and total P (TP) were determined by the Olsen and Dean method [20]. Available potassium (AK) and total K (TK) were measured by the atomic absorption spectroscopy method. NH_4_-N was extracted by 1M KCl solution and determined by an automated discrete analyzer (CleverChem 380, Hamburg, Germany). Soil organic matter (SOM) and total nitrogen (TN) were measured using the K_2_Cr_2_O_7_-H_2_SO_4_ oxidation and the semi-micro-Kjeldahl digestion methods, respectively. The detailed measurement descriptions of the above analyses all followed ISSCAS guidelines [20].

Soil protease activity was measured by the method of Ladd and Butler [21]. Dehydrogenase activity was measured according to the method in ISSCAS guidelines [22]. Polyphenol oxidase activity was measured using the method described by Perucci et al. [23]. Soil alkaline phosphomonoesterase activity was determined using the method described by Schinner et al. [24]. Soil urease activity was determined by using urea as a substrate, and released ammonium was determined by colorimetry at 460 nm [25]. Soil P fractions were determined using the sequential extraction method described by Hedley et al. [26] with some modifications [27]. The P conversion potential of soil microorganisms was estimated by the culture method, which was proposed by Zheng and Zhang [28].

### 2.4. Amplification, Quantification, and Sequencing of phoD and gcd Genes

The soil genomic DNA from NMG, PP, UF, and PF sites was extracted by the Soil DNA Quick Extraction Kit (Bioteke, Wuxi City, China). The fragment of the *phoD* gene was amplified by PCR with the primer pair F1-TGGGAYGATCAYGARGT and R1-CTGSGCSAKSACRTTCCA [29], and the *gcd* gene was amplified by PCR with the primer pair F2-CGGCGTCATCCGGGSITIYRAYRT and R2-GGGCATG TCCATGTCC [30]. The real-time quantitative PCR of the two genes was performed using a real-time PCR system (Q5, Applied Biosystems, Waltham, MA, USA) with SYBR Green as fluorescent dye. Purified PCR products were sequenced on the Illumina MiSeq platform (Shanghai Personal Biotechnology Co., Ltd., Shanghai, China). The operational taxonomic units (OTUs) with 97% similarity cutoff were clustered. The representative sequences of each OTU were taxonomically classified using the BLAST algorithm-based search within GenBank (http://blast.ncbi.nlm.nih.gov/Blast.cgi, accessed on 21 June 2023). The OTU richness was analyzed by the Mothur software (https://www.mothur.org, version 1.21.1, accessed on 21 June 2022). The Simpson diversity index, Shannon–Wiener index, Pielou index, Chao’s species richness estimator (Chao 1), and observed species were calculated [31].

### 2.5. Data Analysis

Soil physical and chemical properties, enzymatic activities, fractions, P conversion rates, and the abundance of *phoD* and *gcd* genes from different sites were statistically analyzed by one-way ANOVA and multiple comparisons followed by an LSD test. The Pearson coefficients between the gene abundance and soil properties, enzymatic activities, P fractions, and P conversion rates were calculated, respectively. All statistical analyses were performed using the SPSS software package (version 18.0). *p <* 0.05 was considered statistically significant. Unweighted pairwise cluster analysis was used to compare the difference in the structure of *phoD* or *gcd* community. LDA effect size (LEfSe) was used to analyze the differentiated taxa among different land-use sites. The significance of the effects of soil factors on *phoD* and *gcd* community structure was determined by redundancy analysis (RDA) performed using the CANOCO 4.5 software (Biometris-Plant Research International, Wageningen, The Netherlands) to determine the main soil factors significantly affecting the two microbial communities, and the correlations of the soil properties were examined by a Monte Carlo permutation. All *phoD* and *gcd* gene sequences were deposited to the NCBI Sequence Read Archive with the accession number of PRJNA734297.

## 3. Results

### 3.1. Soil Properties, P Fractions, and Potential of P Transformation

The results of soil physicochemical properties (including SM, pH, EC, SOM, NH_4_-N, AP, AK, TN, TP, and TK) and enzymatic activities (protease, dehydrogenase, polyphenoloxidase, alkaline phosphomonoesterase, urease, and nitrate reductase) of different sites are shown in Table 1. Except for SM, significant differences in all soil physicochemical properties among different sites were observed (*p* < 0.01). Overall, reforestation in meadow grassland can facilitate the accumulation of soil nutrients, and SOM, AK, TN, and TP in the PP sites were significantly higher than that in UF, PF, and NMG, which were 1.89 to 2.37, 5.93 to 10.30, 5.28 to 6.33, and 1.21 to 2.42 times larger than the other sites, respectively. PF had the lowest SOM, pH, AK, TN, TP, and TK, but soil AP and NH_4_-N were significantly higher than the other sites. Soil AP was sensitive to land-use alternation; the values in PP, UF, and PF were 3.22, 1.81, and 4.51 times higher than that in NMG, respectively.

Meanwhile, the activities of the assayed seven soil enzymes also significantly varied with land-use type (*p* < 0.05). Similar to soil nutrients, all enzymatic activities except for dehydrogenase in the PP site were significantly higher than those in the other sites. The activities of soil protease, polyphenoloxidase, alkaline phosphomonoesterase, urease, and nitrate reductase in the PP site were 3.03 to 8.90, 1.11 to 11.83, 1.26 to 6.35, 1.52 to 15.18, and 3.28 to 31.96 times larger than the other three sites. However, NMG had the highest dehydrogenase activity, and the activities of most soil enzymes in the PF site were all at the lowest levels. Except for soil urease and nitrate reductase, the activities of all the soil enzymes significantly decreased during the conversion from NMG to cropland (UF and PF).

**Figure 2:**
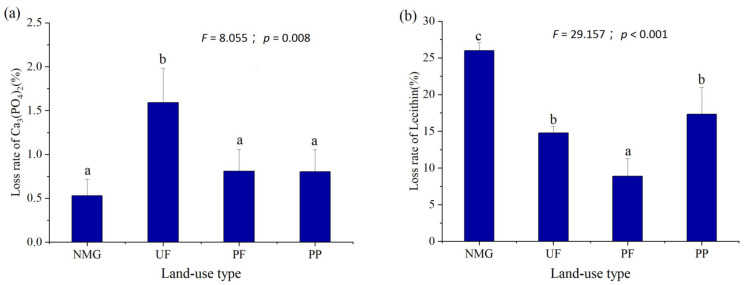
Solubilization potentials of soil inorganic P and mineralization potentials of soil organic P of different sites. NMG: native meadow grassland; UF: 20-year upland field; PF: 20-year paddy field; PP: 20-year poplar plantation. (**a**): solubilization potentials of soil inorganic P estimated by the loss rate of phosphorite; (**b**): mineralization potentials of soil organic P estimated by lecithin quantitatively added in specific microbial medium after incubation for 21 days. *F* and *p* values from one-way ANOVA are given. Means in column followed by different letters are significantly different according to LSD test (*p* < 0.05).

Continuous fractional extraction of soil P was performed, and the results are shown in Table 2. Significant differences in NaHCO_3_-Pi, 0.1M NaOH-Pi, 0.1M NaOH-Po, 1M HCl-Po, 0.5M NaOH-Pi, 0.5M NaOH-Po, residual P, and general P among different sites were found (*p* < 0.05), indicating the difference in the composition of the soil P pool. The available inorganic P fraction (H_2_O-Pi + NaHCO_3_-Pi) was the highest in PF and was 1.45 to 5.06 times higher than that in other sites. The available inorganic P fraction in NMG was at the lowest level. The content of available P stock (H_2_O-Pi + NaHCO_3_-Pi + NaHCO_3_-Po) was the highest in PF, followed by PP, and the lowest in NMG. The general P in the PP and UF sites were significantly higher than that in the other sites. Overall, land-use types had significant effects on different P fractions, residual P, and general P. Significant differences in the loss rates of phosphorite and lecithin added in the medium among different sites were observed (Figure 2, *p* < 0.01). The loss rate of phosphorite ranged from 0.86% in NMG to 1.96% in UF, while the loss rate of lecithin ranged from 8.99% in PF to 25.98% in NMG.

### 3.2. Abundances of gcd and phoD Genes

**Figure 3:**
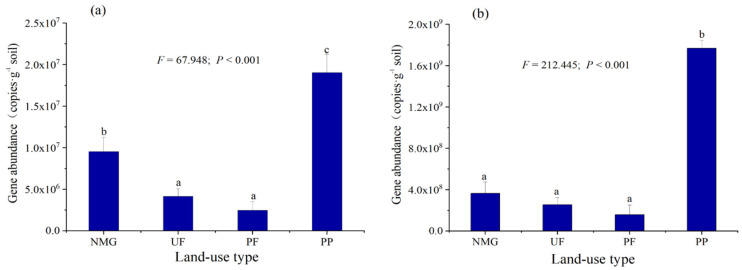
Abundances of soil *gcd* and *phoD* genes at different sites. PP: 20-year poplar plantation; NMG: native meadow grassland; UF: 20-year upland field; PF: 20-year paddy field. (**a**): *gcd* gene; (**b**): *phoD* gene. F and *p* values from one-way ANOVA are given. Means in column followed by different letters are significantly different according to LSD test (*p* < 0.05).

Significant differences in *gcd* and *phoD* gene abundances were observed among different sites. The abundance of the *gcd* gene ranged from 2.47 × 10^6^ copies g^−1^ in the PF site to 1.90 × 10^7^ copies g^−1^ in the PP site, while that of *phoD* ranged from 1.58 × 10^8^ in the PF site to 1.77 × 10^9^ in the PP site (Figure 3, *p* < 0.01). This phenomenon indicated that planting a forest plantation in meadow grassland can significantly increase the quantities of *gcd*- and *phoD*-harboring microbes; however, no significant difference in *phoD* abundance among the sites NMG, UF, and PF and in *gcd* abundance between the sites UF and PF were found. The *gcd* gene abundance was significantly positively correlated with SM, EC, AK, TN, TP, SOM, and the activity of alkaline phosphomonoesterase, with the Pearson coefficients ranging from 0.669 to 0.935, while the abundance of the *phoD* gene was significantly positively correlated with HCl-Po, SM, EC, AK, TP, TN, SOM, the activity of alkaline phosphomonoesterase, 0.1 M NaOH-Po, and 0.5 M NaOH-Po, with the Pearson coefficients ranging from 0.670 to 0.986.

### 3.3. Diversities and Compositions of gcd- and phoD-Harboring Microbial Communities

After Illumina MiSeq high-throughput sequencing, a total of 556182 *gcd* sequences and 353347 *phoD* sequences were obtained from 12 soil samples, and 11549 *gcd* and 7587 *phoD* OTUs were clustered at a 97% cutoff, respectively. The alpha diversity indexes including Chao 1, observed species, Pielou, Simpson, and Shannon–Wiener were calculated and are listed in Table 3. All diversity indexes of *the phoD* community significantly varied with land-use type (*p* < 0.05). Except for Chao1, all alpha diversity indexes of *the phoD* community in NMG were at the lowest level, indicating that the reclamation of meadow grassland induced the increase in the diversity of the *phoD*-harboring community. Meanwhile, the species richness (Chao 1 and observed species) of the *phoD* community in PP was significantly higher than that in NMG, UF, and PF. Similarly, the alpha diversity of the *gcd* community was also significantly altered by land-use type. The transformation of meadow grassland into farmland (UF and PF) resulted in a significant decrease in the richness of the species (Chao1 and Observed species) of *gcd* communities. However, the Chao1 and Observed species in the PP site were significantly higher than those in NMG, UF, and PF.

**Figure 4:**
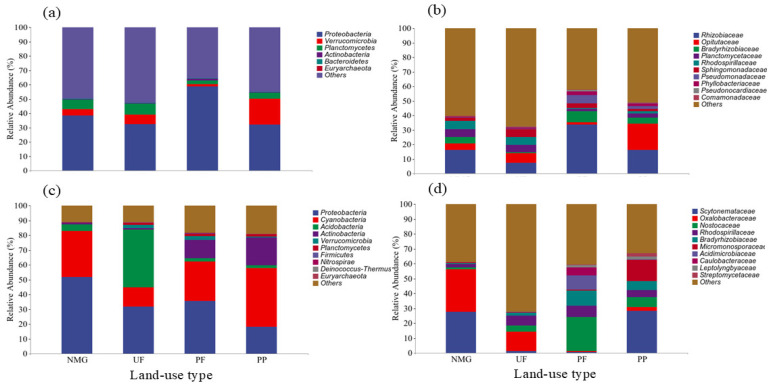
Relative abundances of dominant phyla and families of *gcd* and *phoD* communities in different sites (NMG: native meadow grassland; UF: 20-year upland field; PF: 20-year paddy field; PP: 20-year poplar plantation). (**a**): *gcd* phylum; (**b**): *gcd* family; (**c**): *phoD* phylum; (**d**): *phoD* family.

All detected *gcd* OTUs were classified into six phyla, 25 families, or 22 genera. The identified *gcd* phyla included Proteobacteria, Verrucomicrobia, Planctomycetes, Actinobacteria, Bacteroidetes, and Euryarchaeota. Proteobacteria was definitively the most dominant taxa in all samples, with the relative abundance rangeing from 31.99% in PP to 58.74% in PF. The average relative abundances of Verrucomicrobia and Planctomycetes were 7.71% and 5.30%, respectively. The relative abundances of the other phyla including Actinobacteria, Bacteroidetes, and Euryarchaeota were all <1% (Figure 4). There were 35.61% to 49.85% OTUs that could not be classified at the phylum level according to the GenBank database. Rhizobiaceae was definitively the most dominant *gcd* family in all samples, with an average relative abundance of 18.42%, followed by Opitutaceae (7.70%), Bradyrhizobiaceae (4.23%), Planctomycetaceae (3.65%), Rhodospirillaceae (3.32%), Sphingomonadaceae (3.19%), and Pseudomonadaceae (2.04%). Although most *gcd* OTUs could not be identified based on the GenBank database, several dominant genera including *Rhizobium* (5.78%), *Pseudomonas* (1.56%), *Mesorhizobium* (1.34%), and *Azospirillum* (1.01%) were detected.

The obtained *phoD* OTUs were classified into 12 phyla, 62 families, or 41 genera. The dominant *phoD* phyla (relative abundance >1%) included Proteobacteria (34.4%), Cyanobacteria (27.6%), Acidobacteria (11.81%), Actinobacteria (8.31%), Verrucomicrobia (1.37%), and Planctomycetes (1.19%) (Figure 4). Proteobacteria was the most dominant phylum in all samples, with a relative abundance ranging from 18.21% to 51.87%, followed by Cyanobacteria (13.02% to 39.63%). In addition, Actinobacteria in PF (12.36%) and PP (18.87%) and Acidobacteria in UF (38.87%) were also dominant phyla. The relative abundance of dominant *phoD* families varied with land-use type as well. In the NMG site, Scytonemataceae and Oxalobacteraceae were the most dominant families, with relative abundances of 27.59% and 28.65%, respectively, while in the UF site, Oxalobacteraceae had the highest average relative abundance (12.98%), followed by Rhodocyclaceae (6.76%), Nostocaceae (4.06%), and Bradyrhizobiaceae (2.01%). In the PF site, Nostocaceae (22.63%), Rhodospirillaceae (7.62%), Bradyrhizobiaceae (10.29%), Acidimicrobiaceae (9.34%), and Caulobacteraceae (5.32%) were the dominant families. Scytonemataceae (28.49%) was the most dominant family in the PP site, followed by Micromonosporaceae (14.52%), then Nostocaceae (6.80%), Bradyrhizobiaceae (6.08%), and Rhodospirillaceae (4.60%). Although more than 90% of the OTUs could not be classified at the generic level, some dominant *phoD* genera, including *Bradyrhizobium* (9.90%), *Actinoplanes* (4.46%), *Micromonospora* (2.51%), *Dongia* (1.93%), and *Solirubrobacter* (1.24%), were observed (Figure 4).

**Figure 5:**
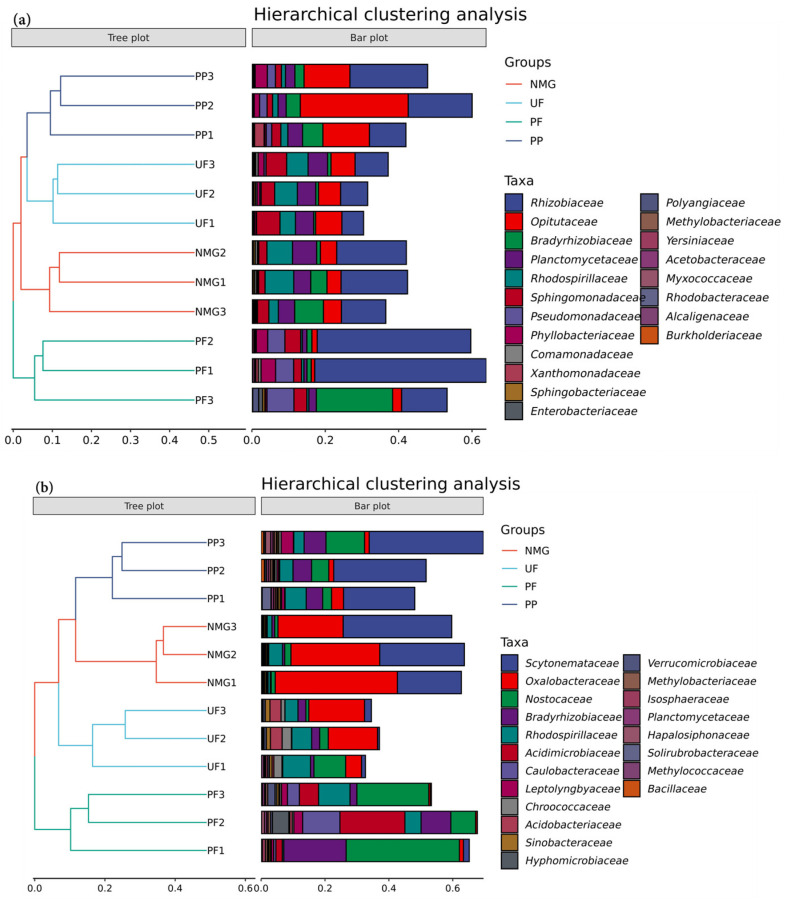
Hierarchical clustering analysis at family level of *gcd* and *phoD* community. (**a**): *gcd* family; (**b**): *phoD* family. NMG: native meadow grassland; UF: 20-year upland field; PF: 20-year paddy field; PP: 20-year poplar plantation.

Hierarchical cluster analysis of different sites was performed based on top 20 dominant families, and the results are shown in Figure 5. All sampling sites were clustered into four groups. The samples from the same site can be *individually* clustered in one group, indicating the differences in the structures of soil *gcd*- and *phoD*-harboring microbial communities.

**Figure 6:**
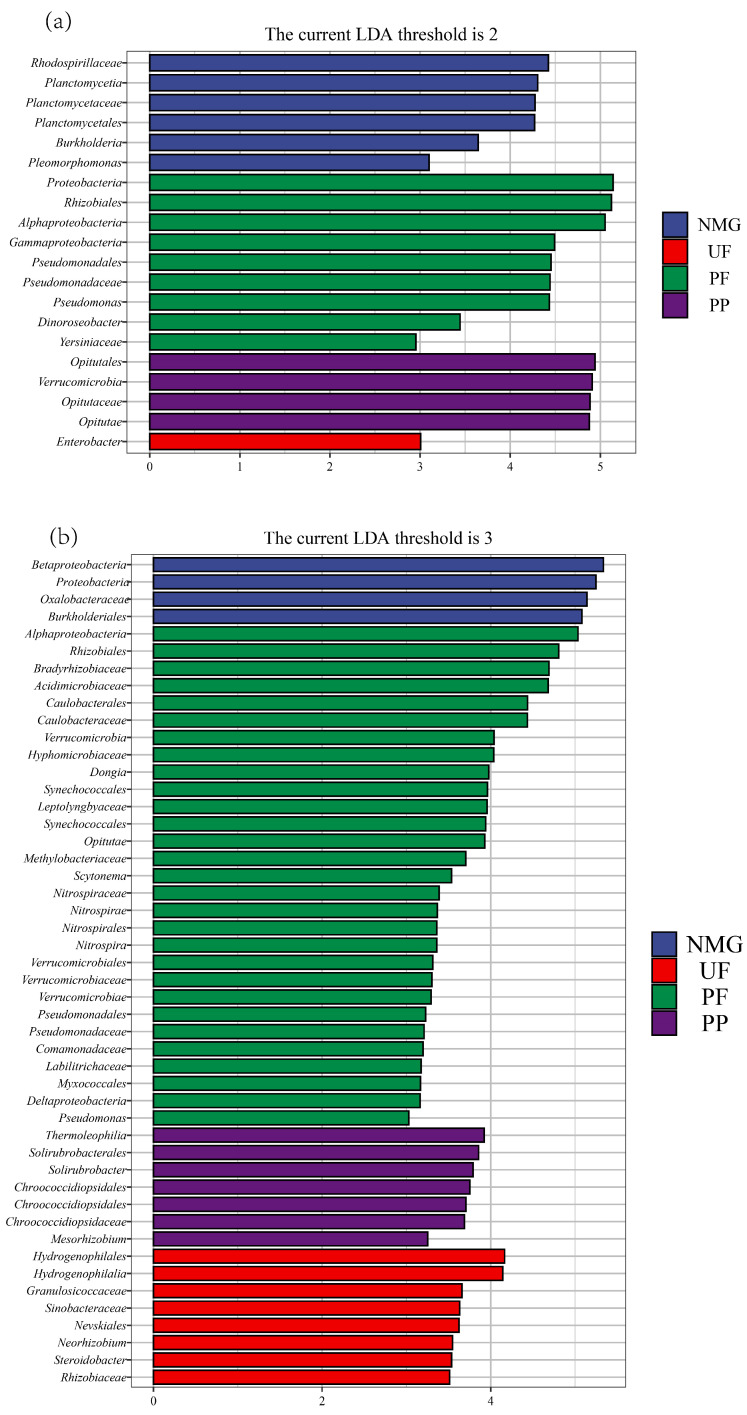
Significantly different taxa detected by LEfSe in *gcd* and *phoD* communities. (**a**): *gcd* taxa; (**b**): *phoD* taxa. A significance level of 0.05 was used for evaluated biomarkers. NMG: native meadow grassland; UF: 20-year upland field; PF: 20-year paddy field; PP: 20-year poplar plantation.

The differentiated taxa in the *gcd* and *phoD* communities among the different sites were further analyzed by the LEfSe method, and the results are shown in Figure 6. In the UF site, the differentiated taxon of the *gcd* community was Enterobacter; in the NMG site, the differentiated taxa included Rhodospirillaceae, Planctomycetia, Planctomycetaceae, Planctomycetales, Burkholderia, and Pleomorphomonas; and in the PP site, they were Verrucomicrobia, Opitutales, Opitutae, and Opitutaceae. PF had more differentiated *gcd* taxa than the other sites, including Proteobacteria, Rhizobiales, Alphaproteobacteria, Gammaproteobacteria, Pseudomonadaceae, *Pseudomonas*, Pseudomonadales, Dinoroseobacter, and Yersiniaceae. The differentiated *phoD*-harboring taxa in the UF site were Hydrogenophilales, Hydrogenophilalia, Granulosicoccaceae, Sinobacteraceae, Nevskiales, Neorhizobium, Steroidobacter, and Rhizobiaceae (Figure 6). In the NMG site, they were Betaproteobacteria, Proteobacteria, Oxalobacteraceae, and Burkholderiales. In the PP site, the differentiated taxa included Thermoleophilia, Solirubrobacterales, Solirubrobacter, Chroococcales, Chroococcaceae, and *Mesorhizobium*. The PF site had more differentiated *phoD* taxa than the other sites, including Alphaproteobacteria, Rhizobiales, Bradyrhizobiaceae, Acidimicrobiaceae, Caulobacterales, Caulobacteraceae, Verrucomicrobia, Hyphomicrobiaceae, Dongia, Synechococcales, Leptolyngbyaceae, Opitutae, Methylobacteriaceae, Scytonema, Nitrospirae, Nitrospira, Nitrospirales, Nitrospiraceae, Verrucomicrobiales, Verrucomicrobiaceae, Verrucomicrobiae, Comamonadaceae, Labilitrichaceae, Myxococcales, Deltaproteobacteria, and *Pseudomonas*. These results further indicate that land-use cover significantly affects the structures of *gcd* and *phoD* communities, which are characterized by significant differences in the relative abundances of the dominant taxa.

### 3.4. Influence of Soil Properties on phoD and gcd Communities

**Figure 7:**
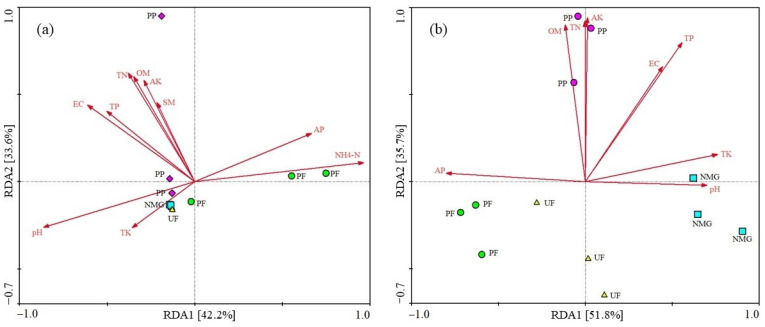
Redundancy analysis (RDA) between the structure of the *phoD* or *gcd* community (top 20 families) and soil properties. (**a**): *gcd* community; (**b**): *phoD* community. The red arrows represented soil properties, and the land use types were differentiated by different colors. OM: organic matter; EC: electrical conductivity; SM: soil moisture; TN: total N; TP: total P; AP: available P; AK: available K; TK: total K. NMG: native meadow grassland; UF: 20-year upland field; PF: 20-year paddy field; PP: 20-year poplar plantation.

RDA was performed to analyze the relationships between the structure of the *gcd* or *phoD* community and soil properties. The results showed that the variations were explained by 42.2% in the first axis and 34.4% in the second axis in the *gcd* community, respectively, while those in the *phoD* community were 51.8% and 35.7%, respectively (Figure 7). Different sites were separately distributed in different quadrants, indicating the difference in the structure of the *phoD* or *gcd* community. Most soil property indicators significantly affected the *gcd* or *phoD* community, of which pH, TN, TP, TK, AP, AK, OM, and EC were the common factors significantly affecting the structure of soil *gcd* and *phoD* communities, and NH_4_-N and SM also significantly affected the *gcd* community structure.

## 4. Discussion

### 4.1. The Influence of Land-Use Type on Soil Properties

During the process of land-use conversion, the changes in vegetation cover and soil attributes depend on the management practices implemented in the conversion process. In the Horqin Sandy Land, the land-use conversion of meadow grasslands usually involves plowing and harrowing the soil and the cultivation of crops or planting trees. This process dramatically altered the soil properties by destroying the vegetation and increasing the rates of organic matter decomposition. Some studies confirmed that the long-term tillage of cropland can modify the soil physicochemical properties and microbiological activity [32], and the addition of phosphate fertilizer reduced microbial biomass and biological activity (e.g., phosphatase activity) [33,34]. Wang et al. [35] concluded that the long-term application of N fertilizer, especially inorganic N, can decrease soil pH and alkaline phosphatase (ALP) activity. However, in a forest system, the leaf litter of plants can effectively increase the accumulation of SOM [36] and plant roots, and root-related microorganisms also make great contributions to the accumulation and storage of SOM [37]. Our results showed that the soil properties, nutrient contents, and enzymatic activities were significantly different among the four land-use types, and PP had the highest nutrient and microbiological activity, while that of two farmlands (UF and PF) were at the lowest level, as the PP or NMG sites are expected to accumulate litter deposits, which are relatively more recalcitrant than the crop debris deposited in agricultural areas [38]. This phenomenon was consistent with the results obtained by Creamer [39], which indicated that land-use systems have significant effects on soil physicochemical properties and microbiological activities. In an agricultural system, a low input of organic matter, variations in temperature and precipitation, and the management of soils were reported to be related to soil properties and the quantity of microorganisms [32].

Different land-uses resulted in significant differences in the fractional composition and availability of soil P, informing that soil P cycling can be regulated by altering land-use types and management levels [40]. The availability of soil P depends on the proportions and contents of different types of P in the P pool which include labile, moderately labile, and non-labile inorganic P (Pi) and organic P (Po) fractions [40,41]. In a natural ecosystem, the labile phosphorus (H_2_O-Pi and NaHCO_3_-Pi) can be directly absorbed and utilized by plants. Moderately labile Pi usually chelates with Fe, Al, and Ca in the soil, while Po is adsorbed by organic matter and soil minerals [26]. The P pool composition of soil was different among different land-use types [40]. In this study, the moderate labile Po in PP was significantly higher than that in UF, PF, and NMG, possibly due to the increased SOM and the increased soil biodiversity, which also increased the amount of easily stabilized P (including H_2_O-Pi, NaHCO_3_-Pi, and NaHCO_3_-Po). Microorganisms can mineralize soil Po by decomposing litter and organic matter through self-secreted phosphatases such as ALP, and the mineralized P can be fixed by microorganisms and then released from the microbial biomass after cell death or left in the soil solution for plant absorption. Soil P responses to land-use cover were mainly mediated by edaphic properties and the amount of P inputs [2,42]. Plant litter and roots can significantly increase P inputs to the soil [43]. And anthropic activities such as land-use conversion can affect the proportion and content of soil P components [44]. Urea addition can stimulate transformation from non-labile Pi to moderately labile Pi and then to labile Pi [45]. Our results also showed that the managed cropland had greater positive effects on labile Pi, NaOH-Pi, and residual P than other P fractions. The results are consistent with the findings of a study in Northwest China by Wang et al. [40], which concluded that irrigation and fertilization increased total P and labile and moderately labile P fractions in topsoil.

### 4.2. The Responses of Soil gcd and phoD Microbial Communities to Land-Use Type

Some previous studies have used microbial *gcd* and *phoD* genes as major molecular markers of soil P transformation function [18,46]. In this study, the abundance of soil *gcd* and *phoD* showed significant differences among different land-use types, indicating the differences in potential abilities of soil Pi dissolution and Po mineralization. Therefore, the abundance of *phoD* and *gcd* genes could be used as an indicator for estimating the bioavailability of soil P. In this study, PP had the highest abundances of *gcd* and *phoD* genes, while the croplands (UF and PF) were at the lowest level, suggesting that PP establishment increased soil P conversion potential; meanwhile, the reclamation of grassland cropland decreased the potential. Turner and Haygarth [47] proposed that ALP was a key enzyme in regulating organic phosphate mineralization. Our results showed that ALP activity and *phoD* gene abundance showed the same varying trends in different land-use types. These results further confirmed that *phoD* is the most common gene coding ALP in soil [16]. In addition, the abundance of the *phoD* gene in soil was about one hundred times more than that of the *gcd* gene in each land-use type, and the rate of Po mineralization was significantly higher than that of Pi solubilization, which illustrates that the availability of soil P mainly depends on the functions of *phoD* microorganisms.

Ragot et al. [48] found that *phoD* is ubiquitous and diverse in soil. In this study, Proteobacteria, Cyanobacteria, Acidobacteria, Actinobacteria, Scytonemataceae, Oxalobacteraceae, Nostocaceae, and Rhodospirillaceae were all the dominant *phoD*-harboring taxa in the four land-use types. The results were similar to some previous studies [49,50]. Although the relative abundance of dominant taxa in the *gcd* and *phoD* communities varied with land-use type, the dominant compositions of *gcd* and *phoD* communities under different types were similar. This phenomenon indicated that the basic compositions of dominant taxa in the same soil type were relatively stable and less affected by the land use or soil properties, probably because of the widespread existence of many core populations which have highly resilient and resistant capacities and are insensitive to environmental variations [51]. Therefore, we infer that the basic compositions of *phoD* and *gcd* communities may mainly depend on local climate or soil type. Turley et al. [52] reported that the basic compositions of soil microbial communities had a close relationship with land-use history. In addition, we observed that some N-fixing bacterial taxa, e.g., Rhizobiaceae, Bradyrhizobiaceae, and *Mesorhizobium*, also carried the *phoD* or *gcd* gene. Similarly, Kalayu [53] reported that *Rhizobium* is an efficient P solubilizer for increasing the bioavailability of P in soil. This phenomenon suggests that these taxa have regulatory functions both on the N cycle and P transformation [49]. Therefore, to further understand the mechanism of soil P transformation, the microbial communities associated with soil N cycle and P transformation functions and the transcription/expression levels of their specific genes should be simultaneously analyzed in future studies. Soil properties are direct factors affecting microbial community structure. In this study, RDA results indicated that soil properties significantly affect the structure of *gcd* and *phoD* communities (Figure 7). pH, TN, TP, TK, AP, AK, OM, and EC were the common factors affecting the structure of soil *gcd* and *phoD* communities, while NH_4_-N and SM only had a significant influence on *gcd* community structure. Cheng et al. [50] reported that pH, AP, and NH_4_-N had a significant influence on the structure of soil *phoD* and *gcd* communities. Higher levels of SOM, TN, AP, and TP in soil were associated with greater *gcd* abundance [54]. The application of organic and biofertilizer promoted the processes of P solubilization [55]. Some previous research found that the long-term application of inorganic N fertilizer resulted in soil acidification and decreased the diversity of *phoD*-harboring bacterial communities [34,35]. Ragot et al. [56] also concluded that pH, TN, SOM, and TP were important soil factors responsible for the structure of *phoD*-harboring communities. Our results confirm that land-use conversion directly alters soil properties, subsequently affecting the quantity and structures of the microbial communities related to organic P mineralization and inorganic P hydrolysis, thereby inducing changes in ALP activity, the rate of P-turnover, and soil AP content.

## 5. Conclusions

In this study, we found that land-use types had significant influences on soil physicochemical properties, enzymatic activities, and P conversion rates, thereby changing the structures of soil *gcd* and *phoD* microbial communities. The abundance of *phoD* and *gcd* genes varied with land-use type and could be used as indicators for estimating the bioavailability of soil P. Soil *phoD* microbes are more abundant and give more contributions to AP than *gcd* microbes. The absolute dominant *gcd* taxa included Proteobacteria, Verrucomicrobia, Planctomycetes, Rhizobiaceae, and Opitutaceae, while the *phoD* community was dominated by the phyla of Proteobacteria, Cyanophyta, Acidobacteria, Actinobacteria, and Verrucomicrobia. The responses of *gcd* or *phoD* communities to land-use cover were characterized as changes in the relative abundance of dominant taxa; however, the basic compositions of the two communities were slightly affected. Soil properties significantly affected the *gcd* and *phoD* communities. Soil pH, EC, and nutrient contents (including organic matter and total and available N, P, and K) all significantly affected soil *gcd* and *phoD* microbial communities. Further studies on the dynamics of soil *phoD* and *gcd* microbial communities and their potential effects on the ecological functions of meadow grassland should be conducted to evaluate the sustainability of land-use systems.

## Figures and Tables

**Table 1:** Soil properties and enzymatic activities at different sites.

Items	NMG	UF	PF	PP	*F*	*p*
SM	2.89 ± 1.12 a	2.01 ± 1.47 a	2.82 ± 0.54 a	4.72 ± 0.39 a	4.048	0.051
pH	8.49 ± 0.28 b	8.35 ± 0.10 b	7.16 ± 0.70 a	7.85 ± 0.05 ab	7.52	0.01
EC	227.7 ± 18.2 b	218.8 ± 69.4 b	136.4 ± 8.4 a	334.3 ± 17.5 c	14.33	0.001
AK	86.1 ± 3.8 ab	106.0 ± 24.1 b	61.0 ± 10.1 a	628.5 ± 4.6 c	1239.8	<0.001
AP	2.91 ± 0.51 a	5.28 ± 1.48 a	13.11 ± 1.21 c	9.38 ± 1.28 b	29.056	0.004
NH_4_-N	2.27 ± 0.35 a	1.32 ± 0.03 a	7.01 ± 3.02 b	2.65 ± 0.45 a	8.089	0.008
TP	0.38 ± 0.006 b	0.19 ± 0.001 a	0.19 ± 0.005 a	0.46 ± 0.008 c	2072.1	<0.001
TK	1.82 ± 0.02 b	1.73 ± 0.02 a	1.72 ± 0.01 a	1.75 ± 0.03 a	8.562	0.007
TN	0.036 ± 0.004 a	0.048 ± 0.008 a	0.030 ± 0.009 a	0.190 ± 0.034 b	53.24	<0.001
SOM	1.05 ± 0.07 a	1.32 ± 0.19 a	1.20 ± 0.19 a	2.51 ± 0.31 b	94.62	<0.001
PRA	152.2 ± 48.0 a	51.9 ± 6.82 a	119.1 ± 73.6 a	461.7 ± 112.2 b	19.48	<0.001
DEA	629.4 ± 29.0 c	211.0 ± 43.5 ab	77.9 ± 17.8 a	351.7 ± 144.1 b	28.08	<0.001
POA	2.44 ± 0.06 b	0.23 ± 0.142 a	0.38 ± 0.140 a	2.72 ± 0.52 b	66.77	<0.001
ALP	72.6 ± 3.98 b	25.8 ± 7.39 a	14.1 ± 4.92 a	91.4 ± 7.80 c	105.5	<0.001
URA	133.0 ± 71.3 b	374.9 ± 13.4 c	37.5 ± 4.6 a	569.3 ± 26.8 d	115.5	<0.001
NRA	0.110 ± 0.023 a	0.224 ± 0.087 a	0.023 ± 0.017 a	0.735 ± 0.511 b	4.693	0.036

Values are means ± SD. NMG: native meadow grassland; UF: 20-year upland field; PF: 20-year paddy field; PP: 20-year poplar plantation. SM: soil moisture (%); EC: electrical conductivity (μs·cm^−1^); AK: available potassium (mg·kg^−1^); AP: available P (mg·kg^−1^); TP: total P (%); TK: total K (%); TN: total N (%); SOM: soil organic matter (%); PRA: protease (μg·Tyr.g^−1^·2 h^−1^); DEA: dehydrogenase (mg·kg^−1^·h^−1^); POA: polyphenol oxidase (μmol·g^−1^·10 min^−1^); ALP: alkaline phosphatase (mg·g^−1^·h^−1^); URA: urease (mg·kg^−1^·24 h^−1^); NRA: nitrate reductase (μg·g^−1^·24 h^−1^). *F* and *p* values from one-way ANOVA are given. Means in the row followed by a different letter are significantly different (*p* < 0.05).

**Table 2:** Soil P fractions (mg·kg^−1^) at different sites.

Fraction	NMG	UF	PF	PP	*F*	*p*
H_2_O-Pi	2.00 ± 0.27 a	9.17 ± 9.34 a	5.09 ± 0.84 a	7.44 ± 3.17 a	1.18	0.377
NaHCO_3_-Pi (0.5 mol·L^−1^)	6.02 ± 0.08 a	17.47 ± 9.58 b	35.53 ± 4.34 c	20.61 ± 5.46 b	12.63	0.002
NaHCO_3_-Po (0.5 mol·L^−1^)	4.55 ± 0.57 a	5.63 ± 1.53 a	9.87 ± 3.97 a	7.78 ± 0.45 a	3.595	0.066
NaOH-Pi (0.1 mol·L^−1^)	2.70 ± 0.20 a	5.06 ± 0.99 b	26.22 ± 1.74 d	19.25 ± 1.09 c	293.3	<0.001
NaOH-Po (0.1 mol·L^−1^)	20.8 ± 3.25 a	19.2 ± 1.04 a	24.7 ± 1.17 a	79.1 ± 25.06 b	15.56	0.001
HCl-Pi (1.0 mol·L^−1^)	20.0 ± 1.00 a	64.3 ± 54.01 a	28.3 ± 5.40 a	20.5 ± 1.26 a	1.802	0.225
HCl-Po (1.0 mol·L^−1^)	27.6 ± 7.32 a	57.0 ± 36.05 a	28.4 ± 11.69 a	77.2 ± 9.57 a	4.374	0.042
NaOH-Pi (0.5 mol·L-1)	3.02 ± 0.38 a	7.71 ± 0.73 b	8.22 ± 1.13 b	12.29 ± 0.20 c	86.96	<0.001
NaOH-Po (0.5 mol·L-1)	27.9 ± 6.44 a	37.9 ± 7.12 a	22.0 ± 8.54 a	70.0 ± 7.99 b	24.03	<0.001
Residual-P	484.3 ± 57.5 a	1579.0 ± 111.0 c	816.0 ± 20.9 b	1523.7 ± 20.2 c	210.9	<0.001
General-P	596.9 ± 51.3 a	1793.3 ± 129.1 c	999.2 ± 30.1 b	1830.4 ± 56.0 c	190.1	<0.001

Values are means ± SD. NMG: native meadow grassland; UF: 20-year upland field; PF: 20-year paddy field; PP: 20-year poplar plantation. *F* and *p* values from one-way ANOVA are given. Means in row followed by different letters are significantly different according to LSD test (*p* < 0.05).

**Table 3:** Diversity indices of *phoD* and *gcd* microbial communities of different land uses.

Index	Gene	NMG	UF	PF	PP	*F*	*p*
Chao1	*phoD*	1241.2 ± 100.9 a	1421.9 ± 279.2 a	1139.1 ± 191.0 a	2425.1 ± 154.6 b	28.183	<0.001
	*gcd*	2885.3 ± 244.3 c	2235.6 ± 216.4 b	1650.5 ± 114.6 a	3541.6 ± 225.8 d	46.901	<0.001
Observed species	*phoD*	652.2 ± 60.5 a	828.0 ± 179.1 a	815.3 ± 154.6 a	1323.2 ± 94.9 b	14.726	0.001
	*gcd*	2029.1 ± 160.6 c	1487.8 ± 213.6 b	1099.1 ± 72.0 a	2616.2 ± 21.0 d	67.861	<0.001
Pielou	*phoD*	0.460 ± 0.036 a	0.599 ± 0.040 b	0.651 ± 0.045 b	0.625 ± 0.006 b	17.251	0.001
	*gcd*	0.758 ± 0.014 b	0.733 ± 0.011 b	0.649 ± 0.044 a	0.740 ± 0.054 b	5.548	0.023
Shannon– Wiener	*phoD*	4.30 ± 0.38 a	5.81 ± 0.58 b	6.29 ± 0.61 b	6.48 ± 0.07 b	13.602	0.002
	*gcd*	8.32 ± 0.09 b	7.72 ± 0.26 b	6.56 ± 0.50 a	8.40 ± 0.62 b	12.356	0.002
Simpson	*phoD*	0.845 ± 0.041 a	0.935 ± 0.017 b	0.948 ± 0.019 b	0.923 ± 0.021 b	9.443	0.005
	*gcd*	0.986 ± 0.004 b	0.981 ± 0.004 b	0.924 ± 0.032 a	0.977 ± 0.023 b	6.197	0.018

Values are means ± SD. NMG: native meadow grassland; UF: 20-year upland field; PF: 20-year paddy field; PP: 20-year poplar plantation. *F* and *p* values from one-way ANOVA are given. Means in row followed by different letters are significantly different by LSD test (*p* < 0.05).

## Data Availability

The original contributions presented in the study are included in the article; further inquiries can be directed to the corresponding author.

## References

[1] Cui F.Q., Wang B.J., Zhang Q., Tang H.P., De Maeyer P., Hamdi R., Dai L.W. (2021). Climate change versus land-use change—What affects the ecosystem services more in the forest-steppe ecotone?. Sci. Total Environ..

[2] Li F.R., Liu J.L., Ren W., Liu L.L. (2018). Land-use change alters patterns of soil biodiversity in arid lands of northwestern China. Plant Soil.

[3] Paltineanu C., Dumitru S., Vizitiu O., Mocanu V., Lăcătusu A.R., Ion S., Domnariu H. (2024). Soil organic carbon and total nitrogen stocks related to land use and basic environmental properties−assessment of soil carbon sequestration potential in different ecosystems. Catena.

[4] Zhu R.H., Azene B., Gruba P., Pan K., Nigussie Y., Guadie A., Sun X.M., Wu X.G., Zhang L. (2024). Response of carbohydrate-degrading enzymes and microorganisms to land use change in the southeastern Qinghai-Tibetan Plateau, China. Appl. Soil Ecol..

[5] Liu S.L., Sun Y.X., Shi F.N., Liu Y.X., Wang F.F., Dong S.K., Li M.Q. (2022). Composition and diversity of soil microbial community associated with land use types in the agro-pastoral area in the upper Yellow River Basin. Front. Plant Sci..

[6] Wang J.C., Zou Y.K., Di G.D., Singh B.K., Li Q.F. (2020). Conversion to agroforestry and monoculture plantation is detrimental to the soil carbon and nitrogen cycles and microbial communities of a rainforest. Soil Biol. Biochem..

[7] Vitousek P.M., Porder S., Houlton B.Z., Chadwick O.A. (2010). Terrestrial phosphorus limitation: Mechanisms, implications, and nitrogen-phosphorus interactions. Ecol. Appl..

[8] Condron L.M., Turner B.L., Cademenun B.J., Sims J.T., Sharpley A.N. (2005). Chemistry and dynamics of soil organic phosphorus. Phosphorus Agric. Environ..

[9] Mahdi I., Fahsi N., Hafidi M., Allaoui A., Biskri L. (2020). Plant growth enhancement using rhizospheric halotolerant phosphate solubilizing bacterium Bacillus licheniformis QA1 and Enterobacter asburiae QF11 isolated from Chenopodium quinoa willd. Microorganisms.

[10] Mendoza-Arroyo G.E., Chan-Bacab M.J., Aguila-Ramírez R.N., Ortega-Morales B.O., Canché Solís R.E., Chab-Ruiz A.O., Cob-Rivera K.I., Dzib-Castillo B., Tun-Che R.E., Camacho-Chab J.C. (2020). Inorganic phosphate solubilization by a novel isolated bacterial strain *Enterobacter* sp. ITCB-09 and its application potential as biofertilizer. Agriculture.

[11] Nannipieri P., Giagnoni L., Landi L., Renella G., Else B., Astrid O., Emmanuel F. (2011). Role of Phosphatase Enzymes in Soil. Phosphorus in Action.

[12] Li J.B., Xie T., Zhu H., Zhou J., Li C.N., Xiong W.J., Xu L., Wu Y.H., He Z.L., Li X.Z. (2021). Alkaline phosphatase activity mediates soil organic phosphorus mineralization in a subalpine forest ecosystem. Geoderma.

[13] Fraser T., Lynch D.H., Entz M.H., Dunfield K.E. (2015). Linking alkaline phosphatase activity with bacterial *phoD* gene abundance in soil from a long-term management trial. Geoderma.

[14] Kageyama H., Tripathi K., Rai A.K., Cha-Um S., Waditee-Sirisattha R., Takabe T. (2011). An alkaline phosphatase/phosphodiesterase, phoD, induced by salt stress and secreted out of the cells of *Aphanothece halophytica*, a halotolerant cyanobacterium. Appl. Environ. Microbiol..

[15] Wu J.R., Shien J.H., Shieh H.K., Hu C.C., Gong S.R., Chen L.Y., Chang P.C. (2007). Cloning of the gene and characterization of the enzymatic properties of the monomeric alkaline phosphatase (PhoX) from *Pasteurella multocida* strain X-73. FEMS Microbiol. Lett..

[16] Tan H., Barret M., Mooij M.J., Rice O., Morrissey J.P., Dobson A., Griffiths B., O’Gara F. (2013). Long-term phosphorus fertilization increased the diversity of the total bacterial community and the *phoD* phosphorus mineralizer group in pasture soils. Biol. Fertil. Soils.

[17] Zeng Q.W., Wu X.Q., Wen X.Y. (2016). Effects of soluble phosphate on phosphate-solubilizing characteristics and expression of *gcd* gene in *Pseudomonas frederiksbergensis* JW-SD2. Curr. Microbiol..

[18] Li Y., Zhang J.Q., Gong Z.L., Xu W.L., Mou Z.S. (2019). *Gcd* gene diversity of quinoprotein glucose dehydrogenase in the sediment of Sancha Lake and its response to the environment. Int. J. Environ. Res. Public Health.

[19] Cao C.Y., Jiang D.M., Teng X.H., Jiang Y., Liang W.J., Cui Z.B. (2008). Soil chemical and microbiological properties along a chronosequence of *Caragana microphylla* Lam. plantations in the Horqin Sandy Land of Northeast China. Appl. Soil Ecol..

[20] ISSCAS (Institute of Soil Science, Chinese Academy of Sciences) (1978). Physical and Chemical Analysis Methods of Soils.

[21] Ladd J.N., Butler J.H.A. (1972). Short-term assays of soil proteolytic enzyme activities using proteins and dipeptide derivatives as substrates. Soil Biol. Biochem..

[22] ISSCAS (Institute of Soil Science, Chinese Academy of Sciences) (1985). Methods on Soil Microorganisms Study.

[23] Perucci P., Casucci C., Dumontet S. (2000). An improved method to evaluate the odiphenol oxidase activity of soil. Soil Biol. Biochem..

[24] Schinner F., Öhlinger R., Kandeler E., Margesin R. (1997). Methods in soil biology. J. Ecol..

[25] Kandeler E., Gerber H. (1988). Short-term assay of soil urease activity using colorimetric determination of ammonium. Biol. Fertil. Soils.

[26] Hedley M.J., Stewart J.W.B., Chauhan B.S. (1982). Changes in inorganic and organic soil phosphorus fractions induced by cultivation practices and by laboratory incubations. Soil Sci. Soc. Am. J..

[27] Da Silva V.M., Antoniolli Z.I., Jacques R.J.S., Ott R., Rodrigues P.E.D., Andrade F.V., Passos R.R., Mendonca E.D. (2017). Influence of the tropical millipede, *Glyphiulus granulatus* (Gervais, 1847), on aggregation, enzymatic activity, and phosphorus fractions in the soil. Geoderma.

[28] Zheng H.Y., Zhang D.S. (1982). Methods on Dynamics of Soil Biochemistry Study.

[29] Chen X.D., Jiang N., Condron L.M., Dunfield K.E., Chen Z.H., Wang J.K., Chen L.J. (2019). Impact of long-term phosphorus fertilizer inputs on bacterial *phoD* gene community in a maize field, Northeast China. Sci. Total Environ..

[30] Bergkemper F., Kublik S., Lang F., Kruger J., Vestergaard G., Schloter M., Schulz S. (2016). Novel oligonucleotide primers reveal a high diversity of microbes which drive phosphorous turnover in soil. J. Microbiol. Methods.

[31] Schloss P.D., Westcott S.L., Ryabin T., Hall J.R., Hartmann M., Hollister E.B., Lesniewski R.A., Oakley B.B., Parks D.H., Robinson C.J. (2009). Introducing Mothur: Opensource, platform independent, community-supported software for describing and comparing microbial communities. Appl. Environ. Microbiol..

[32] Hartmann M., Frey B., Mayer J. (2015). Distinct soil microbial diversity under long-term organic and conventional farming. ISME J..

[33] Xie Y.Y., Wang F.H., Wang K., Yue H.Z., Lan X.F. (2020). Responses of bacterial *phoD* gene abundance and diversity to crop rotation and feedbacks to phosphorus uptake in wheat. App. Soil Ecol..

[34] Wang M., Wu Y., Zhao J., Liu Y., Chen Z., Tang Z. (2022). Long-term fertilization lowers the alkaline phosphatase activity by impacting the *phoD*-harboring bacterial community in rice-winter wheat rotation system. Sci. Total Environ..

[35] Wang L., Zhang H., Xu C., Yuan J., Xu X.J., Wang J.D., Zhang Y.C. (2023). Long-term nitrogen fertilization and sweet potato cultivation in the wheat-sweet potato rotation system decrease alkaline phosphomonoesterase activity by regulating soil phoD-harboring bacteria communities. Sci. Total Environ..

[36] Zhang Y.J., Zou J.L., Meng D.L., Dang S.N., Zhou J.H., Osborne B., Ren Y.Y., Liang T., Yu K.K. (2020). Effect of soil microorganisms and labile C availability on soil respiration in response to litter inputs in forest ecosystems: A meta-analysis. Ecol. Evol..

[37] Clemmensen K.E., Bahr A., Ovaskainen O., Dahlberg A., Ekblad A., Wallander H., Stenlid J., Finlay R.D., Wardle D.A., Lindahl B.D. (2023). Roots and associated fungi drive long-term carbon sequestration in boreal forest. Science.

[38] Lauber C.L., Ramirez K.S., Aanderud Z., Lennon J., Fierer N. (2013). Temporal variability in soil microbial communities across land-use types. ISME J..

[39] Creamer R.E., Hannula S.E., Van Leeuwen J.P., Stone D., Rutgers M. (2016). Ecological network analysis reveals the inter-connection between soil biodiversity and ecosystem function as affected by land use across Europe. Appl. Soil Ecol..

[40] Wang Z.R., Li F.R., Liu L.L., Yang K. (2022). Changes in soil phosphorus fractions under different land uses in desert grasslands in northwestern China. Eur. J. Soil Sci..

[41] García-Velázquez L., Rodríguez A., Gallardo A., Maestre F., Santos E., Lafuente A., Fernández M., Singh B., Wang J., Durán J. (2020). Climate and soil micro-organisms drive soil phosphorus fractions in coastal dune systems. Funct. Ecol..

[42] Chen L.F., He Z.B., Zhao W.Z., Liu J.L., Zhou H., Li J., Meng Y.Y., Wang L.S. (2019). Soil structure and nutrient supply drive changes in soil microbial communities during conversion of virgin desert soil to irrigated cropland. Eur. J. Soil Sci..

[43] Feng J., He K., Zhang Q., Han M., Zhu B. (2022). Changes in plant inputs alter soil carbon and microbial communities in forest ecosystems. Glob. Change Biol..

[44] Zhang Y.Q., Bhattacharyya R., Dalal R.C., Wang P., Menzies N.W., Kopittke P.M. (2020). Impact of land use change and soil type on total phosphorus and its fractions in soil aggregates. Land Degrad. Dev..

[45] Wang R.Z., Liu H.Y., Sardans J., Feng X., Xu Z.W., Peñuelas J. (2021). Interacting effects of urea and water addition on soil mineral bound phosphorus dynamics in semi-arid grasslands with different land-use history. Eur. J. Soil Sci..

[46] Hu M.J., Penuelas J., Sardans J., Tong C., Chang C.T., Cao W.Z. (2020). Dynamics of phosphorus speciation and the *phoD* phosphatase gene community in the rhizosphere and bulk soil along an estuarine freshwater-oligohaline gradient. Geoderma.

[47] Turner B.L., Haygarth P.M. (2005). Phosphatase activity in temperate pasture soils: Potential regulation of labile organic phosphorus turnover by phosphodiesterase activity: Linking landscape sources of phosphorus and sediment to ecological impacts in surface waters. Sci. Total Environ..

[48] Ragot S.A., Kertesz M.A., Bünemann E.K. (2015). *phoD* alkaline phosphatase gene diversity in soil. Appl. Environ. Microbiol..

[49] Luo G.W., Ling N., Nannipieri P., Chen H., Raza W., Wang M., Guo S.W., Shen Q.R. (2017). Long-term fertilisation regimes affect the composition of the alkaline phosphomonoesterase encoding microbial community of a vertisol and its derivative soil fractions. Biol. Fertil. Soils.

[50] Cheng J., Zhang Y., Wang H.N., Cui Z.B., Cao C.Y. (2022). Sand-fixation plantation type affects soil phosphorus transformation microbial community in a revegetation area of Horqin Sandy Land, Northeast China. Ecol. Eng..

[51] Suleiman A.K.A., Manoeli L., Boldo J.T. (2013). Shifts in soil bacterial community after eight years of land-use change. Syst. Appl. Microbiol..

[52] Turley N.E., Bell-Dereske L., Evans S.E., Brudvig L.A., Yang G.W. (2020). Agricultural land-use history and restoration impact soil microbial biodiversity. J. Appl. Ecol..

[53] Kalayu G. (2019). Phosphate solubilizing microorganisms: Promising approach as biofertilizers. Int. J. Agron..

[54] Zhang L.C., Ren G., Chu G.X. (2023). Land reclamation increased labile and moderately labile P fractions and strengthened co-occurrence network of *gcd* community in calcareous soils. Land Degrad. Dev..

[55] Du T.Y., Hu Q.F., Mao W.J., Yang Z., Chen H., Sun L.N., Zhai M.Z. (2023). Metagenomics insights into the functional profiles of soil carbon, nitrogen, and phosphorus cycles in a walnut orchard under various regimes of long-term fertilisation. Eur. J. Agron..

[56] Ragot S.A., Huguenin-Elie O., Kertesz M.A., Frossard E., Bunemann E.K. (2016). Total and active microbial communities and *phoD* as affected by phosphate depletion and pH in soil. Plant Soil.

